# Identification of actinomycetes from plant rhizospheric soils with inhibitory activity against *Colletotrichum *spp., the causative agent of anthracnose disease

**DOI:** 10.1186/1756-0500-4-98

**Published:** 2011-04-01

**Authors:** Bungonsiri Intra, Isada Mungsuntisuk, Takuya Nihira, Yasuhiro Igarashi, Watanalai Panbangred

**Affiliations:** 1 Department of Biotechnology, Faculty of Science, Mahidol University, Bangkok 10400, Thailand; 2 MU-OU Collaborative Research Center for Bioscience and Biotechnology, Faculty of Science, Mahidol University, Bangkok 10400, Thailand; 3 Center of Excellence for Agricultural Biotechnology (AG-BIO), Postgraduate Education and Research Development Office, Commission on Higher Education, Ministry of Education, Thailand; 4 International Center for Biotechnology, Osaka University, Osaka 565-0871, Japan; 5 Biotechnology Research Center, Toyama Prefectural University, Toyama 939-0398, Japan

## Abstract

**Background:**

*Colletotrichum *is one of the most widespread and important genus of plant pathogenic fungi worldwide. Various species of *Colletotrichum *are the causative agents of anthracnose disease in plants, which is a severe problem to agricultural crops particularly in Thailand. These phytopathogens are usually controlled using chemicals; however, the use of these agents can lead to environmental pollution. Potential non-chemical control strategies for anthracnose disease include the use of bacteria capable of producing anti-fungal compounds such as actinomycetes spp., that comprise a large group of filamentous, Gram positive bacteria from soil. The aim of this study was to isolate actinomycetes capable of inhibiting the growth of *Colletotrichum *spp, and to analyze the diversity of actinomycetes from plant rhizospheric soil.

**Results:**

A total of 304 actinomycetes were isolated and tested for their inhibitory activity against *Colletotrichum gloeosporioides *strains DoA d0762 and DoA c1060 and *Colletotrichum capsici *strain DoA c1511 which cause anthracnose disease as well as the non-pathogenic *Saccharomyces cerevisiae *strain IFO 10217. Most isolates (222 out of 304, 73.0%) were active against at least one indicator fungus or yeast. Fifty four (17.8%) were active against three anthracnose fungi and 17 (5.6%) could inhibit the growth of all three fungi and *S. cerevisiae *used in the test. Detailed analysis on 30 selected isolates from an orchard at Chanthaburi using the comparison of 16S rRNA gene sequences revealed that most of the isolates (87%) belong to the genus *Streptomyces *sp., while one each belongs to *Saccharopolyspora *(strain SB-2) and *Nocardiopsis *(strain CM-2) and two to *Nocardia *(strains BP-3 and LK-1). Strains LC-1, LC-4, JF-1, SC-1 and MG-1 exerted high inhibitory activity against all three anthracnose fungi and yeast. In addition, the organic solvent extracts prepared from these five strains inhibited conidial growth of the three indicator fungi. Preliminary analysis of crude extracts by high performance liquid chromatography (HPLC) indicated that the sample from strain JF-1 may contain a novel compound. Phylogenetic analysis revealed that this strain is closely related to *Streptomyces cavurensis *NRRL 2740 with 99.8% DNA homology of 16S rRNA gene (500 bp).

**Conclusion:**

The present study suggests that rhizospheric soil is an attractive source for the discovery of a large number of actinomycetes with activity against *Colletotrichum *spp. An interesting strain (JF-1) with high inhibitory activity has the potential to produce a new compound that may be useful in the control of *Colletotrichum *spp.

## Background

Anthracnose disease caused by species of *Colletotrichum *is one of the most economically important plant diseases and is responsible for reducing the marketable yield of crop production (10% to 80% reduction) in some developing countries such as Thailand, Pakistan, Turkey and Mexico [1,2]. *Colletotrichum *spp. can cause anthracnose disease in fruits such as avocado, guava, papaya, mango and passion fruit [3]. Two significant *Colletotrichum *species found in Thailand are *C. capsici *and *C. gloeosporioides *and these same pathogens are also the main phytopathogens in South America and Asia, especially tropical regions of Asia [4]. *C. capsici *generally infects mature fruit, while *C.gloeosporioides *infects both green and mature fruits [4].

Although phytopathogens are usually suppressed using synthetic chemicals, the excessive use of chemical control has led to environmental pollution. Moreover, the efficiency of these pesticides is continually decreasing due to the development of resistant pathogens. Over the last 25-30 years, improvement of alternative control methods, including the use of microorganisms, has been undertaken [4]. Most biological controls are directed toward wound pathogens and involve the use of antagonistic bacteria that produce antibiotics [5-7].

Actinomycetes, particularly *Streptomyces *species are among the richest sources of antibiotics [8-10]. Roughly 60% of biologically active compounds that have been developed for agricultural use originated from *Streptomyces*[11]. Various groups of bioactive compounds such as macrolide, benzoquinones [12], aminoglycosides [13,14], polyenes [15,16], and nucleoside antibiotics [17,18] are examples of agriculturally useful metabolites produced from *Streptomyces*.

In order to find bioactive compounds that may be useful in the control of fungal pathogens, such as *Colletotrichum *spp., we isolated actinomycetes species and screened them for antifungal activity. Of the actinomycetes isolated, 30 selected strains were also subjected to phylogenetic analysis using comparison of their16S rRNA gene sequences to investigate the diversity of actinomycetes in rhizospheric soil collected from orchards.

## Results and Discussion

### Isolation of actinomycetes

A total of 304 actinomycetes isolates were obtained from 39 rhizospheric soil samples collected from orchards in Chanthaburi, Bangkok, Petchaburi, and Nongbualamphu provinces. The collection areas included the eastern, central and northeastern parts of Thailand. Rhizospheric soil was selected for use in this study since rhizosphere-associated soils can contain almost twice as many actinomycetes isolates as non-rhizosphere-associated soils, according to a previous report by Crawford and co-workers [19].

Initially, isolated colonies had a smooth appearance but later developed a weft of aerial mycelium that either appeared floccose, granular, powdery or velvety as described in Bergey's Manual of Determinative Bacteriology [20]. Colonies of actinomycetes, in particular those of *Streptomyces *species, were picked on the basis of their morphological characteristics on agar plates. The *Streptomyces *colonies can be easily identified by their opaque, rough, nonspreading morphology and are usually embedded resulting in adherence to agar medium. The color of substrate and aerial mycelia was variable; however, almost any colony gave an earthy odor that is characteristic of *Streptomyces*.

### Antimicrobial activity of actinomycete isolates

Among the 304 actinomycetes isolates, 214 (70.4%), 110 (36.2%), 64 (21.1%), 42 (13.8%) displayed activity against the growth of *C. gloeosporioides *strains DoA d0762 and DoA c1060, *C. capsici *strain DoA c1511 and *S. cerevisiae *strain IFO 10217, respectively. Two hundred and twenty two isolates (73.0%) were active against at least one indicator microorganism. Inhibition toward *C. gloeosporioides *strain DoA d0762 was shown by 214 isolates, and inhibition toward *C. capsici *strain DoA c1511 was shown by 64 isolates. Fifty four strains (17.8%) inhibited all of three fungi which caused anthracnose disease. Only 17 isolates (5.6%) inhibited all indicator fungi as well as yeast. The use of several indicator microorganisms helped to select strains with potent activity [11,17]. Antifungal activity of the 304 actinomycete isolates is summarized in Table 1.

**Table 1:** Summary of antifungal activity of the 304 actinomycete isolates by the co-culture method

Total number of isolated strains against indicator fungi											
A*	B*	C*	D*	**A+B**	**A+C**	**A+D**	**B+C**	**B+D**	**C+D**	**A+B+C**	**A+B+C+D**

214 70.4%	110 36.2%	64 21.1%	42 13.8%	109 35.9%	59 19.4%	35 11.5%	58 19.1%	25 8.2%	19 6.3%	54 17.8%	17 5.6%

*A is *C. gloeosporioides *strain DoA d0762; B is *C. gloeosporioides *strain DoA c1060; C is *C. capsici *strain DoA c1511 and D is *Saccharomyces cerevisiae *strain IFO10217

### Molecular phylogeny of the 30 selected isolates based on 16S rRNA gene

To assess the actinobacterial diversity in rhizospheric soils from orchards, 30 isolates from a Chanthaburi orchard were selected for additional analysis. The orchard contains several types of fruit trees including rambutan, jack fruit, longkong, lychee, and salak. The orchard was selected due to the variety of fruit trees since differences in tree and fruit materials can affect diversity of the soil microbes [21-23]. The nucleotide sequences for a section of the 16S rRNA gene (500 bp) from the 30 selected strains (GenBank Accession numbers GU13002-GU130031) were subjected to BLASTN analysis using the NCBI database for identification at the genus level. All 30 isolates contained different nucleotide sequences for the 16S rRNA gene, indicating that they were different strains (data not shown). Of the actinomycetes tested 26 out of 30 strains belonged to genus *Streptomyces *and contained between 95-100% DNA homology in the 16S rRNA gene. Two strains (strains BP-3 and LK-1) had shown proximity to genus *Nocardia*. Strains SB-2 and CM-2 belong to genera *Saccharopolyspora *and *Nocardiopsis*, respectively (Table 2). The prevalence of *Streptomyces *species over other actinomycetes was likely due to screening conditions (media and cultivation). Pridham and water proline agar media used in this study are preferred by *Streptomyces*[24].

**Table 2:** Antifungal activity of 30 actinomycete isolates obtained from rhizospheric soil samples in Chanthaburi

Soil samples collected under	Designated number	Closely related strain*	Antifungal activity**
Rambutan	RB-1	*S. longisporus *strain: NBRC 12885 (AB184219.1)	A
Black pepper	BP-1	*S. bikiniensis *strain WANG-1 (EU560974.1)	-
	BP-2	*Streptomyces* sp. MJM5732 (EU603355.1)	A+B
	BP-3	*Nocardia araoensis* strain DSM 44729 isolate VNS41 (AY903623.1)	A+B
Longkong	LK-1	*Nocardia thailandica *(AB126874.1)	A+B
Lychee	LC-1	*S. griseocarneus *strain: NBRC 13428 (AB184863.1)	A+B+C+D
	LC-2	*S. spororaveus *strain HBUM174519 (EU77063.1)	A+B
	LC-3	*S. lavendulae *strain IFO 3125 (D85106.1)	D
	LC-4	*S. herbaricolor *strain NRRL B-3299T (DQ442505.2)	A+B+C+D
Leech lime	LL-1	*Streptomyces *sp. 210726 (EU600089.1)	-
	LL-2	*Streptomyces *sp. A00032 (EF690247.1)	-
Cumin	CM-1	*S. antibioticus *strain: NBRC 13271 (AB184340.1)	A
	CM-2	*Nocardiopsis *sp. 94N10-1 (EU196477.1)	A+B
Jack fruit	JF-1	*S. cavourensis *subsp. *cavourensis *strain NRRL 2740 (DQ445791.1)	A+B+C+D
Siam cardamom	SC-1	*S. triostinicus *strain CKM7 (EU635725.1)	A+B+C+D
Dragon fruit	DF-1	*S. collinus *subsp. *albescens *strain NBRC 12547 (AB184101.2)	A
Pisang mas	PM-1	*S. aureus *strain HBUM174596 (EU841581)	A
	PM-2	*S. roseocinereus *isolate S55-4 (EU521698.1)	A+B
	PM-3	*S. roseocinereus* isolate S55-4 (EU521698.1)	A+B
	PM-4	*S. setonensis *strain NBRC 13797 (AB184488.1)	-
	PM-5	*S. cinnamocastaneus *strain HBUM173422 (EU841658)	A
Tamarind	TM-1	*S. pseudovenezuelae *strain IMER-B1-8 (FJ796459.1)	-
	TM-2	*S. pseudovenezuelae *strain IMER-B1-8 (FJ796459.1)	A
Sapodilla	SP-1	*S. triostinicus *strain CKM7 (EU635725.1)	A+B
	SP-2	*S. triostinicus *strain CKM7 (EU635725.1)	-
Spiny bamboo	SB-1	*S. triostinicus *strain CKM7 (EU635725.1)	A+B
	SB-2	*Saccharopolyspora gregorii *strain 4037-02-0614 (AY588271)	A
Cassumanar	CS-1	*S. paradoxus *strain HBUM174056 (EU841653)	A
	CS-2	*S. bikiniensis *strain 13661V (EU741193)	A+B
Mango	MG-1	*S. lividus *strain NBRC 13787 (AB184480.1)	A+B+C+D

* Identified by partial 16S rRNA gene sequence comparison, only the highest similarity was shown.

**Designation of A, B, C and D were the same as shown in Table 1.

**Figure 1:**
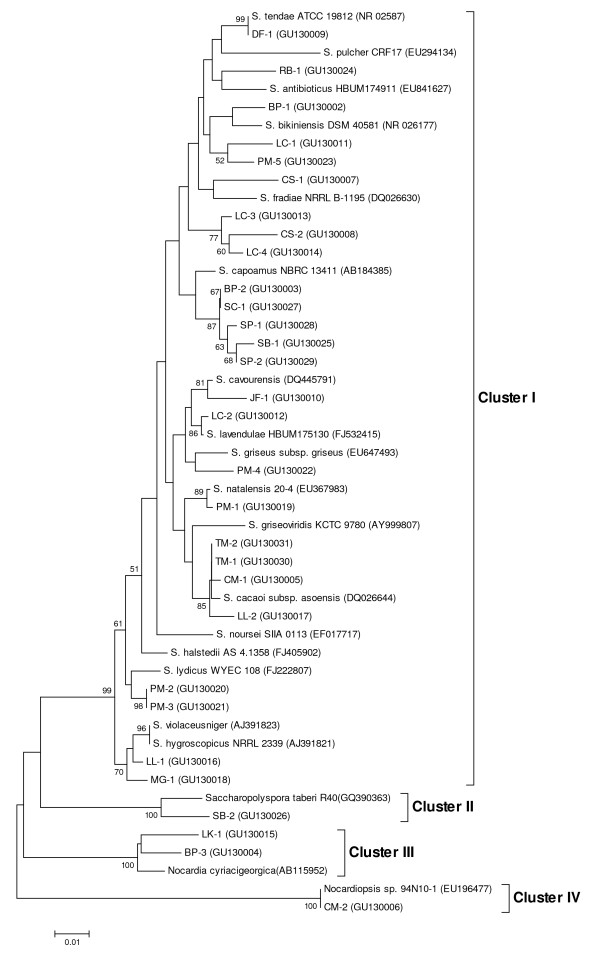
**Neighbor-joining tree based on partial sequences of 16S rDNA (500 bp) of 30 selected isolates**. The numbers at the nodes indicate percentages of bootstrap support (n = 1,000 re-samplings). Bootstrap values less than 50% are not shown. The scale bar corresponds to 0.01 nucleotide substitution per site.

The results described above were supported by phylogenetic analysis based on the neighbor-joining tree. The 30 isolates were sorted into 4 main clusters with highest similarity to the genera *Streptomyces*, *Saccharopolyspora*, *Nocardia *and *Nocardiopsis*, respectively (Figure 1). The BP-3 and LK-1 isolates matched with high sequence identity to members of the *Nocardia cyriacigeorgica *clade, while SB-2 and CM-2 isolates were closely related to *Saccharopolysporaterberi *R40 and *Nocardiopsis *sp. 94N10-1 clade members, respectively. Most of the selected isolates (26 strains) were closely matched to *Streptomyces *spp. Our results suggest that actinomycetes in plant rhizospheric soils from orchards are diverse and that these strains are suitable for natural product screening.

### Determination of antifungal activity of 5 potent antagonistic Streptomyces strains

The bioassay results for the 30 selected actinomycetes strains against the three pathogens and yeast identified 24 strains with antimicrobial potential especially against *C. gloeosporioides *DoA d0762 (Table 2). Furthermore, 5 *Streptomyces *strains (LC-1, LC-4, JF-1, SC-1, and MG-1) were found to be active against all indicator microorganisms (Table 2) and they were classified in distinct branches from each other (Figure 1). A previous report has indicated that taxonomically related strains might have some similarity in their metabolic pathways though certain strains were unique in their metabolic profiles [25]. Therefore these five strains, which are clustered in different groups, might produce different compounds with antifungal properties.

These five *Streptomyces *isolates were also active against conidial germination. As indicated in Table 3, they had antagonistic activity against *C. gloeosporioides *strains DoA d0762 and DoA c1060, and *C. capsici *strain DoA c1511. A clear zone, indicating inhibition of growth, ranging from 28-64 mm in diameter appeared around the colonies of the five isolates against the growth of *C. gloeosporioides *strain DoA d0762. Isolates JF-1 and MG-1 showed strong inhibition against three fungal strains with the inhibition zone ranging from 30 to 41 mm in diameter for *C. gloeosporioides *strain DoA c1060 and *C. capsici *strain DoA c1511. JF-1 and MG-1 displayed even stronger activity against the *C. gloeosporioides *strain DoA d0762 with an inhibition zone of greater than 60 mm in diameter.

**Table 3:** Antifungal activity of five *Streptomyces *isolates by the co-culture method against fungal conidial suspensions

Isolates	Activity against (mm)		
	
	*C.gloeosporioides* DOA d0762	*C.gloeosporioides* DOA c1060	*C. capsici* DOA c1511
LC-1	35.5	15.5	18
LC-4	28	20	21.5
JF-1	60.5	35.5	35.5
SC-1	30.5	17	10.5
MG-1	64	30.5	38.5

The antifungal activity was measured after incubation for 7 days at 28°C and the distance indicated the zone of inhibition against the germination of indicator microorganisms. The inhibition zone included the diameter (8 mm) of the *Streptomyces *agar block.

**Figure 2:**
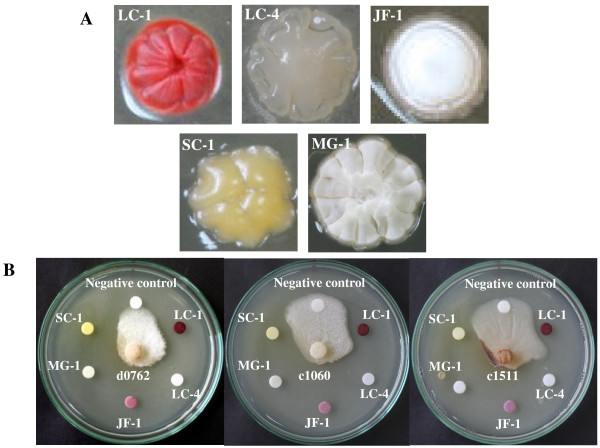
**Colony morphology of five selected *Streptomyces *isolates (strains LC-1, LC-4, JF-1, SC-1 and MG-1) (A) and activity of their crude extracts against *C. gloeosporioides *DoA d0762, *C. gloeosporioides *DoA c1060 and *C. capsici *DoA c1511 (B)**. In (A), colony morphology of the five strains (LC-1, LC-4, JF-1, SC-1 and MG-1) was observed on Waksman agar after seven days of growth. In (B), five paper disks (6 mm in diameter) loaded with 20 μl of crude extracts were plated at a 2 cm distance around fungal agar block (8 mm in diameter) which was placed at the center of the plate. The negative control was a disk loaded with 20 μl of ethanol. All discs were completely dried before placing on the plate.

**Figure 3:**
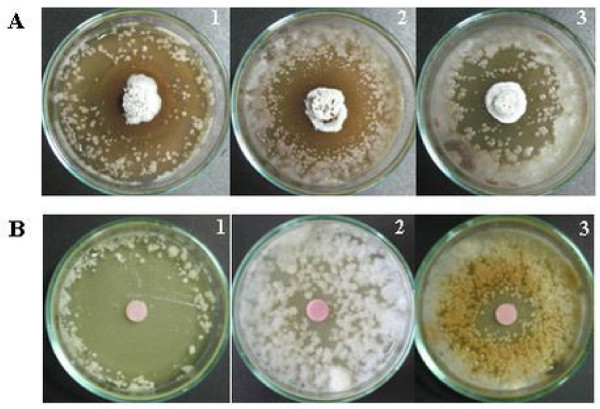
**Antifungal activity of *Streptomyces *JF-1 (A) and its crude extract (B) against conidial suspensions of *C. gloeosporioides *DoA d0762 (A-1 and B-1), *C. gloeosporioides *DoA c1060 (A-2 and B-2) and *C. capsici *DoA c1511 (A-3 and B-3)**. The results were observed at day 7.

The mechanism of antifungal antagonists can be due to the secretion of hydrolytic enzymes such as chitinase, β,3 glucanase, chitosanase, and proteases [26] which degrade the fungal cell wall, or the secretion of antifungal compounds [27]. *Streptomyces *spp. from rhizospheric soil can protect roots by inhibiting the development of potential fungal pathogens; however, it is not known whether the zone of inhibition, caused by the five *Streptomyces *strains, we isolated occurs as a result of extracellular hydrolytic enzymes or antifungal metabolites or the combined action of both. To determine whether the 5 selected isolates (LC-1, LC-4, JF-1, SC-1, and MG-1) with high antifungal activity produced antifungal metabolites, crude extracts were prepared by solvent extraction. Using a disk diffusion susceptibility test inhibition of mycelial growth was clearly observed in the presence of cell extracts after incubation for 7 days. It is possible that these strains are producing antifungal metabolites, since activity was retained in solvent extracted preparations [22,23]. Colony morphology of the five selected *Streptomyces *(strain LC-1, LC-4, JF-1, SC-1 and MG-1) and activity of their crude extracts against *C. gloeosporioides *strain DoA c1060 are illustrated in Figure 2A and 2B, respectively. All five crude extract samples inhibited mycelial growth of the three anthracnose fungi (Figure 2B). Crude extract of MG-1 and JF-1 exerted a higher inhibitory activity over the other three crude extract samples as shown by larger inhibition distance as well as shorter outgrowth of fungal mycelia from the fungal agar block (Figure 2B). The antifungal activity of JF-1 colony as well as its crude extract against conidial growth of *Colletotrichum *spp. is shown in Figure 3A and 3B. Its crude extract shows strong inhibition against conidial suspension of *C. gloeosporioides *strains DoA d0762 (Figure 3, B-1).

### HPLC-DAD (High-Performance Liquid Chromatography with Diode-Array Detection) analysis

Crude extracts from the 5 isolates were separated by HPLC and analyzed for their retention times and UV-visible properties. The results show that crude extracts from 3 strains (LC-4, SC-1, and MG-1) produced known compounds; strain LC-1 produced no significant peaks; however, the JF-1 isolate appeared to produce novel compounds (Table 4). The novelty of the secondary metabolites from strain JF-1 remains to be confirmed through purification and structural elucidation of the metabolites and these compound(s) are currently under investigation. From the analysis of the HPLC profiles it appears that the five isolates, which reside in different phylogenetic branches (Figure 1), produce different bioactive compounds (Table 4).

**Table 4:** HPLC analysis of crude extracts

Isolates	Peaks at retention time (min)	Remarks
SC-1	22.5 (240, 440; actinomycin)	known
LC-1	No significant peaks	no peak
LC-4	6.2 (220, 270; Trp-containing peptide)	known
JF-1	24.0 (250, 285), 25.9 (230)	unknown
MG-1	10.4 (290, 330; staurosporin)	known

Note: The numbers in parenthesis indicate the wavelength in nm

## Conclusions

In summary, plant rhizospheric soil contains abundant actinomycetes species capable of producing different antifungal compounds. HPLC analysis of cell extracts revealed that one of the isolated strains (JF-1) has a high potential to produce novel bioactive compounds that were not present in our database. Purification and structure determination are essential for the further analysis and application of these compounds for use in the control of *Colletotrichum *and anthracnose disease.

## Methods

### Indicator microorganisms and culture conditions

*Colletotrichum gloeosporioides *strains DoA d0762 and DoA c1060 and *C. capsici *strain DoA c1511 were obtained from Department of Agriculture, Ministry of Agriculture and Co-operatives, Thailand. *Saccharomyces cerevisiae *strain IFO 10217 was kindly provided by Dr. Chuenchit Boonchird, Department of Biotechnology, Faculty of Science, Mahidol University, Thailand. The fungi and yeast were incubated at 28°C and maintained on potato dextrose agar (PDA) or 301 medium (2.4% starch, 0.1% glucose, 0.5% yeast extract, 0.3% meat extract, 0.4% CaCO_3_, and 1.2% agar).

### Isolation of actinomycetes from soil samples

Rhizospheric soils (39 samples) were collected from under fruit trees or plants in fruit orchards from Chanthaburi, Bangkok, Petchaburi, and Nongbualamphu provinces. Soil samples (1 g) were suspended in normal saline solution (9 ml). The suspension was serially diluted to final dilutions of 10^-4^, 10^-5 ^and 10^-6^. Aliquots (0.1 ml) of each dilution were spread on Pridham's agar (Pr) (1% glucose, 1% starch, 0.2% (NH_4_)_2_SO_4_, 0.2% CaCO_3_, 0.1% K_2_HPO_4_, 0.1% MgSO_4_, 0.1% NaCl and 1.2% agar) and Water-proline agar (WA) (1% praline and 1.2% agar) supplemented with 25 μg/ml nalidixic acid and 50 μg/ml cycloheximide to prevent growth of other bacteria and fungi, respectively. Plates were incubated at 28°C for 4-14 days. Isolated actinomycetes were further subcultured by incubating at 28°C and maintained on Seino's (1% starch, 0.3% N-Z amine typeA, 0.1% yeast extract, 0.1% meat extract, 0.3% CaCO_3, _and 1.2% agar) and Waksman agar slants (1% glucose, 0.5% peptone, 0.5% meat extract, 0.3% NaCl, and 1.2% agar). The cultures on Seino's or Waksman agar were kept at room temperature and 4°C or -20°C, respectively. Strains were subcultured to fresh media every 2 months. For long-term preservation, conidial or mycelial suspension in 25% glycerol were kept at -80°C.

### Screening of antifungal activity by co-culture method

Antifungal activity was evaluated as described previously [28]. The fungi and actinomycetes were grown on 301 medium at 28°C for 7 days. An agar block of indicator fungus was prepared using a sterile cork borer with diameter of 8 mm, and placed at the center of the plate. Six agar blocks, each block containing a single actinomycete colony, were inoculated around the fungal block at a distance of 10 mm from the edge of the agar block. The plates were incubated at 28°C and inhibition of mycelial growth was measured after 7 days [28]. For testing the inhibitory activity against yeast, *S. cerevisiae *was grown in PDB broth at 30°C for 2 days and a sterile cotton swab was dipped into this culture and spread evenly onto PDA plates. Agar blocks of seven-day-old cultures of isolated actinomycetes were placed on the PDA plate (six isolates/plate). After incubition for 2 days, the inhibition zone was measured. The five strains that showed antifungal activity were further tested for inhibition of conidial germination by inoculating a single agar block of each isolate onto an agar plate (55 mm in diameter) previously spread with fungal conidia (10^5 ^conidia/plate).

### Extraction and bioassay for antifungal activity

Five strains (strain LC-1, LC-4, JF-1, SC-1 and MG-1) showing strong activity against all three indicator fungi were cultivated in 100 ml of 301 broth at 28°C for 2 days in 500-ml Erlenmeyrer flasks. The pre-culture (10 ml) was inoculated into 200 ml of 301 medium in a 500-ml Erlenmeyrer flask, followed by incubation at 28°C for 6 days with rotary shaking at 150 rpm. After the cultivation, an equal volume of absolute ethanol was added to the entire culture. The mixture was shaken at 150 rpm for 45 minutes and the organic layer was obtained by centrifugation. The cell-free supernatant was evaporated to half the original volume and then extracted with equal volume of ethyl acetate. The ethyl acetate-extract was evaporated and the residue was dissolved in ethanol (1 ml). For bioassays, the extracts (about 0.2-0.4 mg/20 μl) were loaded onto a sterile filter disk (diameter 6 mm). The disk was left at room temperature until completely dried. A blank disk loaded with 20 μl of ethanol was used as negative control. The six disks were placed on the surface of agar medium around an agar block (8 mm in diameter) containing each indicator fungus. The inhibition zone was measured after further incubation at 28°C for 7 days.

### HPLC-DAD (High-Performance Liquid Chromatography with Diode-Array Detection) analysis

Analytical HPLC was carried out with a Rainin Microsorb C18 column (4.6 × 75 mm) on an Agilent HP1100 system with a photodiode array detector (200-600 nm). The flow rate was 1.2 ml/min, and additional UV detection was measured at 254 nm. The mobile phase used was a stepwise gradient of CH_3_CN-0.15% KH_2_PO_4 _(pH 3.5) (15%-85% v/v). Chemical library analysis was performed by comparing the UV spectra and retention time of respective peaks to those of compounds in the database at Toyama Prefectural University, Japan.

### 16S rRNA gene sequence analysis for bacterial identification at the genus level

Partial 16S rRNA gene sequences (~500 bp) from the isolates were determined from PCR-amplified fragments. The genomic DNA used as templates for PCR was prepared from single colonies grown for 4-5 days on Waksman agar plate using the boiling method [29]. The 16S rRNA fragment (~500 bp) was amplified using universal primers UFUL (5'-GCCTAACACATGCAAGTCGA-3') and URUL (5'-CGTATTACCGCGGCT GCTGG-3') [30]. The PCR conditions consisted of an initial denaturation at 5 min at 94°C followed by 30 cycles of amplification (94°C for 30 sec, 55°C for 30 sec and 72°C for 30 sec) and an additional 7 min at 72°C. The direct sequencing of PCR products was performed by dideoxy chain termination method using 3100-Avant Genetic Analyzer (Applied Biosystems), USA. The obtained sequences were analyzed for homology using BLASTN [31].

### Phylogenetic analysis of isolates

16S rRNA sequences of 30 strains were aligned with 16S rRNA sequences of other actinomycetes retrieved from the EMBL/GenBank database. Multiple alignments were performed manually using Clustal × [32]. Neighbor-joining phylogenetic tree and molecular evolutionary analyses were conducted using MEGA version 4 [33].

### Nucleotide sequence accession numbers

The nucleotide sequences of 16S rRNA from the 30 isolates investigated in this study have been deposited in the GenBank database library under accession numbers GU130002-GU130031.

## Competing interests

The authors declare that they have no competing interests.

## Authors' contributions

BI collected rhizospheric soil samples, isolated the cultures and tested their antifungal activities, performed the phylogenetic analyses, and wrote the manuscript. YI supervised and performed HPLC-DAD analysis. IL and TN participated on actinomycete isolation and crude extract preparation, respectively. WP was involved in research planning, supervised all analyses and participated in manuscript writing. All authors read and approved the final manuscript.
